# Prevalence and transmission of pretreatment drug resistance and acquired drug resistance among people living with HIV-1 in Chongqing, China, 2014–2024

**DOI:** 10.3389/fpubh.2026.1923017

**Published:** 2026-09-16

**Authors:** Wenwan Gao, Jungang Li, Mei Li, Kun He, Renni Deng, Kun Yang

**Affiliations:** 1 Department of Medical Laboratory Medicine, Chongqing Public Health Medical Center, Chongqing, China; 2 Division of Infectious Diseases, Chongqing Public Health Medical Center, Chongqing, China

**Keywords:** acquired drug resistance, HIV-1, mutation, pretreatment drug resistance, subtype

## Abstract

**Objectives:**

To explore the epidemic trends of pretreatment drug resistance (PDR) and acquired drug resistance (ADR) among people living with HIV (PLWH) from 2014 to 2024 in Chongqing, China.

**Methods:**

Samples and clinical data of PLWH were collected from 2014 to 2024. The HIV-1 pol gene was amplified using One-step reverse transcription-polymerase chain reaction (RT-PCR) and nested PCR. HIV-1 drug resistance and subtype were analyzed by the HIV drug resistance database of Stanford University. Logistic regression analysis was identified the risk factor associated with HIV drug resistance (HIVDR).

**Results:**

In this study, a total 1,299 ART-naive PLWH and 2,700 ART-experienced PLWH were enrolled. The overall prevalence of PDR was 22.47% with initial increases followed by decreases during 2014–2024; the overall prevalence of ADR was 43.63% with a decreasing trend. Among PLWH with PDR and ADR, high-level resistance to lamivudine (3TC) and emtrictitabine (FTC)was prevalent, whereas potential low-level resistance to protease inhibitors (PIs) and integrase strand transfer inhibitor (INSTIs) was predominant. For NNRTIs, a high proportion of potential low-level resistance was observed in patients with PDR; by contrast, nevirapine (NVP) and efavirenz (EFV) showed a high proportion of high-level resistance in the ADR population. Notably, high prevalence of resistance to various antiretroviral therapy (ART) drugs was observed in the dominant HIV-1 CRF07_BC subtype. M184V (NRTI-associated mutation) and V179D (NNRTI-associated mutation) were the most frequently identified observed in PDR and ADR sequences; in contrast, the prevalent drug resistance mutation sites associated with PIs and INSTIs differed between PDR and ADR cases. In CRF07_BC with PDR and ADR, the most prevalent mutations were V179D and M184V, respectively. Furthermore, distinct patterns of drug resistance mutations associated with different ART regimens were observed when comparing PDR and ADR populations. Among ART-naive PLWH, females and CRF08_BC were identified as significant risk factors for HIVDR. In contrast, among ART-treated PLWH, risk factors such as HIV-1 subtypes and ART regimens were associated with HIV drug resistance.

**Conclusion:**

Collectively, these results revealed the distinct epidemiological profiles of PDR and ADR in Chongqing area, underscoring the necessity of sustained, routine surveillance of PDR and ADR, and providing strong support to guide personalized regimen selection and reduce the risk of virological failure.

## Introduction

Antiretroviral therapy (ART) has been the most effective treatment for HIV-1 infection and has substantially reduced HIV-related morbidity and mortality. Globally, 31.6 million people had access to ART in 2024, compared with only 7.7 million in 2010 (1, 2). Transmitted drug resistance (TDR) refers to HIV drug resistance present in individuals with newly acquired HIV infection. And pretreatment drug resistance (PDR) is a broader term that encompasses drug resistance before initiating ART, which can be caused by TDR or acquired during prior treatments. Conversely, acquired drug resistance (ADR) is defined as the emergence of resistance mutations after ART initiation, driven by the selective pressure from incomplete viral suppression. However, the widespread use of ART has been accompanied by the emergence of TDR and ADR, both of which could jeopardize the efficacy and increase the risk of virological failure.

According to WHO's report, the prevalence of PDR and ADR varied substantially across geographical regions (1). In Iran, the ADR prevalence was 34% for nucleoside reverse transcriptase inhibitors (NRTIs), 27% for non-nucleoside reverse transcriptase inhibitors (NNRTIs) and 9% for protease inhibitors (PIs), while the PDR prevalence was 3% for NRTIs, 5% for NNRTIs and 0 for PIs (3). In four African countries (Uganda, Kenya, Tanzania, and Nigeria), the PDR prevalence was 4.7% for NRTIs, 8.2% for NNRTIs and 0.4% for PIs, and the overall prevalence exhibited an increasing trend over time; meanwhile, the ADR prevalence was 83.6% for NNRTIs, 67.8% for NRTIs and 1.8% for PIs (4). Conversely, a large Europe cohort reported the PDR prevalence of 5.6% for NRTIs, 8.2% for NNRTIs and 3.7% for PIs; and the ADR prevalence of 42.2% for NNRTIs, 59.1% for NRTIs and 24.2% for PIs. Notably, the overall PDR and ADR prevalence in Europe demonstrated a decreasing trend (5).

In 2003, China initiated the National Free Antiretroviral Treatment Program which recommended two NRTIs combined with one NNRTI as the preferred, standardized, first-line ART regimen (6). In 2016, the Chinese government further expanded the treatment policy to recommend that all PLWH receive ART therapy (7). Despite these substantial advances in treatment accessibility, a national study reported that the overall PDR prevalence increased from 2.6% during 2004–2006 to 6.9% during 2021–2022, which showed increasing trends all the time (8). A recent study covering 2022–2023 indicated that the PDR and ADR prevalence in China was 6.64% and 44.96% respectively (9). Furthermore, substantial geographical heterogeneity was observed in HIV-1 genetic characteristics as well as PDR and ADR prevalence across China. For instance, the PDR prevalence was 5.7% in Wenzhou, 8.4% in Nanning, 12.9% in Beijing and 23.9% in Liangshan Prefecture (10–13). The ADR prevalence was 27.8% in Beijing, 57.4% in Liangshan Prefecture, 46.1% in Guangzhou (12–14).

Chongqing, located in southwest China, is one of the area most severely affected by HIV/AIDS, with over 110,000 PLWH. Sexual transmission accounts for 98.3% of all HIV infections in this region, and individuals aged over 50 years constitute the majority of newly reported cases (15). Previous studies only focused on PDR or ADR in Chongqing (15, 16), lacking of comprehensive analysis of the epidemiological characteristics and risk factors associated with both PDR and ADR. Driven by socioeconomic development, increased population mobility and updated antiretroviral therapy policies, the distribution of HIV-1 genotypes has become increasingly widespread and complex, further contributing to the diversity of drug resistance. Therefore, this study will meticulously analyze the similarities and differences in the epidemiological characteristics of PDR and ADR in the Chongqing from 2014 to 2024 and explore the associated risk factors, aiming to provide evidence for rational medication guidance and individualized ART optimization.

## Methods

### Study design and participants

The study recruited 7256 PLWH who visited Chongqing Public Health Medical Center between 2014 and 2024. Plasma samples were collected from patients who met the following inclusion criteria. The inclusion criteria: (1) sufficient clinical data; (2) completed testing of CD4+ T cell count and HIV viral load; (3) completed HIV drug resistance testing for NRTIs-, NNRTIs- and PIs drugs. Eligible participants were further divided into the ART-naive group and the ART-treated group according to their prior ART exposure. Due to the absence of data for 2019, the temporal trends in HIVDR prevalence were analyzed across five stratified time intervals: 2014-2015, 2016-2017, 2018-2020, 2021-2022 and 2023-2024.

### Sequencing and HIV-1 subtyping

Viral RNA was extracted from plasma samples using the viral nucleic acid extraction kit (Shuoshi, jiangsu, China) according to the manufacturer's instructions. The targeted 1,500 bp fragment in HIV-1 pol gene, which was the protease and reverse transcriptase region, was amplified using One-step reverse transcription-polymerase chain reaction (RT-PCR) (2029F:5′ TGGAAATGTGGRAAGGAAGGAC3′ and 3529R:5′ GCTAyyAAGTCTTTTGATGGGTCAT3′) and nested PCR (2249F:5′ CTTCCCTCARATCACTCT3′ and 3521R:5′ GTCTTTTGATGGGTCATA3′). The HIV-1 integrase region was amplified by One-step RT-PCR using primers 4063F 5′ TCATTCARGCACAACCAG3′) and 5219R 5′ CCTAGTGGGATGTGTACTTCTGAAC3′). Purified PCR products were subsequently subjected to Sanger sequencing. The sequence was spliced and edited, then submitted to the HIV drug resistance database of Stanford university to determine subtypes.

### Drug resistance analysis

The clinically-relevant resistance to NRTIs, NNRTIs, PIs and INSTIs of the edited sequences were evaluated by the HIV drug resistance database of Stanford university. The resistance levels of each antiretroviral drug were categorized as susceptible, potential low-level resistance, low-level resistance, intermediate resistance, or high-level resistance.

### Statistical analysis

Categorical variables were described as percentages or frequencies. Comparisons among groups were performed using the chi-square test or Fisher exact test. Logistic regression analysis was used to identify risk factor associated with HIVDR. Statistical significance was identified at *P* < 0.05. SPSS 26.0 version was used for all analyses and GraphPad prism 8.0 was performed for graphing.

### Ethics statement

The included HIV-1 pol sequences obtained between 2014 and 2024 in this retrospective study was approved by the Ethic Committee of Chongqing Public Health Center (2024-030-02-KY and 2023-004-01-KY-KT). The informed consents from the included PLWH were waived by the Ethic Committee. The main reason is that Chongqing Public Health Center is the only designated hospital in Chongqing responsible for admitting and treating PLWH, and that PLWH are not required to sign a written informed consent form when undergoing routine genotypic resistance testing and treatment at the hospital. And no personally identifiable information was involved.

## Results

### Characterization of the participants

According to the inclusion criteria, 3,999 PLWH were enrolled including 1,299 ART-naive PLWH and 2,700 ART-treated PLWH (Figure 1). Table 1 showed that among the ART-naive and ART-treated cohorts, the dominated PLWH were males (79.68%; 76.59%), aged ≥50 years (51.12%; 49.04%), had a CD4+ T cell count <200 (79.98%; 73.30%) and harbored HIV viral load > 10E5 (75.52%; 61.70%). For treatment regimens, 3TC+TDF+EFV was the predominant first-line regimen among ART-naive PLWH, accounting for 93.61% of cases. In contrast, TAF+FTC+BIC was the most frequently administered regimen (31.22%) in the ART-treated group. In terms of HIV-1 subtypes, CRF07_BC was the most prevalent subtype in both ART-naive and ART-treated PLWH (56.66% and 54.59%, respectively), followed by CRF01_AE (22.02% and 23.56%). The total PDR and ADR prevalence were 22.47% and 43.63%, respectively. Temporal trend analysis revealed dynamic changes in HIVDR prevalence in Chongqing from 2014 to 2024. The PDR prevalence increased from 8.11% in 2014–2015 to a peak of 24.14% in 2021–2022, and then decreased to 23.77% in 2023–2024. Conversely, the overall ADR prevalence exhibited a continuous downward trend, decreasing from 92.78% in 2014–2015 to 35.17% in 2023–2024. Over the 10 years, substantial decreases were observed in ADR prevalence for NRTIs and NNRTIs. However, the ADR prevalence of PIs drugs increased gradually from 1.03% (2014–2015) to 3.16% (2023–2024). Similarly, the ADR prevalence of INSTIs rose from 0.27% in 2018–2020 to 4.62% in 2023–2024 (Supplementary Table S1).

**Figure 1:**
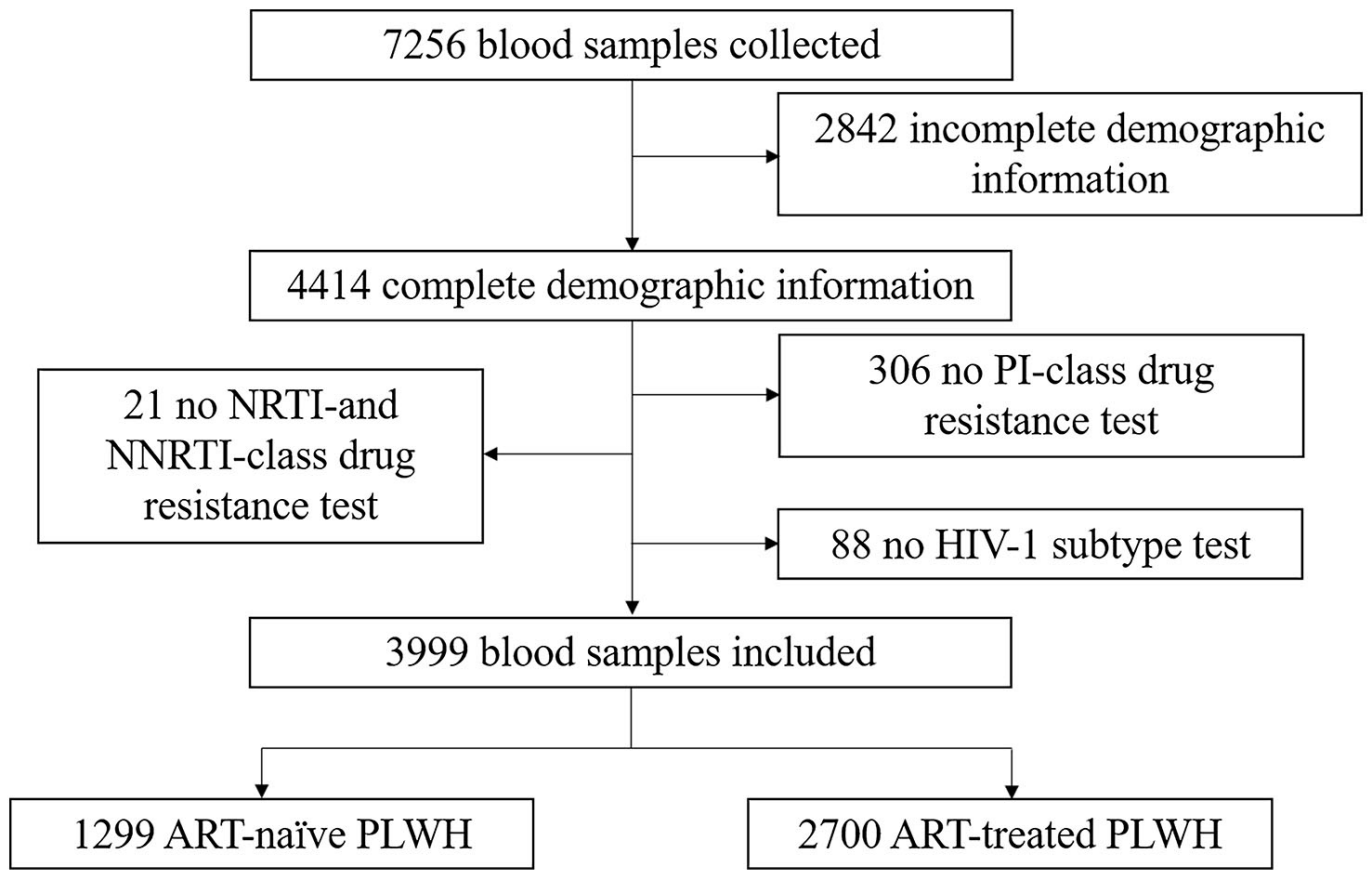
Blood sample collection.

**Table 1:** Characterization of the population.

	ART-naive PLWH						ART-treated PLWH					
Variable	NRTI, *N* (%)	NNRTI, *N* (%)	PI, *N* (%)	INSTI, *N* (%)	PDR, *N* (%)	Total, *N* (%)	NRTI, *N* (%)	NNRTI, *N* (%)	PI, *N* (%)	INSTI, *N* (%)	ADR, *N* (%)	Total, *N* (%)
Sex												
Male	18 (58.06)	188 (76.11)	24 (80.00)	4 (66.67)	219 (75.00)	1,035 (79.68)	459 (78.73)	777 (76.18)	65 (74.71)	60 (72.29)	893 (75.81)	2,068 (76.59)
Female	13 (41.94)	59 (23.89)	6 (20.00)	2 (33.33)	73 (25.00)	264 (20.32)	124 (21.27)	243 (23.82)	22 (25.29)	23 (27.71)	285 (24.19)	632 (23.41)
Age (years)												
≤ 24	3 (9.68)	16 (6.48)	1 (3.33)	1 (16.67)	18 (6.16)	75 (5.77)	40 (6.86)	58 (5.69)	3 (3.45)	3 (3.61)	63 (5.35)	162 (6.00)
25–49	15 (48.39)	125 (50.61)	14 (46.67)	1 (16.67)	143 (48.97)	560 (48.97)	347 (59.52)	525 (51.47)	33 (37.93)	37 (44.58)	590 (50.08)	1,214 (44.96)
≥50	13 (41.94)	106 (42.91)	15 (50.00)	4 (66.67)	131 (44.86)	664 (51.12)	196 (33.62)	437 (42.84)	51 (58.62)	43 (51.81)	525 (44.57)	1,324 (49.04)
CD4+ T cell count (cells/μL)												
<200	29 (93.55)	200 (80.97)	21 (70.00)	4 (66.67)	234 (80.14)	1,039 (79.98)	458 (78.56)	788 (77.25)	59 (67.82)	61 (73.49)	893 (75.81)	1,990 (73.70)
200–500	2 (6.45)	42 (17.00)	8 (26.67)	2 (33.33)	52 (17.81)	232 (17.86)	107 (18.35)	206 (20.20)	27 (31.03)	17 (20.48)	252 (21.39)	634 (23.48)
>500	0 (0.00)	5 (2.02)	1 (3.33)	0 (0.00)	5 (1.71)	28 (2.16)	18 (3.09)	26 (2.55)	1 (1.15)	5 (6.02)	33 (2.80)	76 (2.81)
Viral load level (copies/ml)												
<1,000	0 (0.00)	3 (1.21)	0 (0.00)	0 (0.00)	3 (1.03)	8 (0.62)	19 (3.26)	29 (2.84)	0 (0.00)	5 (6.02)	35 (2.97)	66 (2.44)
1,000–100,000	10 (32.26)	63 (25.51)	8 (26.67)	2 (33.33)	67 (22.95)	310 (23.86)	278 (47.68)	404 (39.61)	36 (41.38)	38 (45.78)	469 (39.81)	968 (35.85)
>100,000	21 (67.74)	181 (73.28)	22 (73.33)	4 (66.67)	218 (74.66)	981 (74.66)	286 (49.06)	587 (57.55)	51 (58.62)	40 (48.19)	674 (57.22)	1,666 (61.70)
HIV subtype												
CRF07_BC	15 (48.39)	103 (41.70)	20 (66.67)	3 (50.00)	130 (44.52)	736 (56.66)	238 (40.82)	424 (41.57)	63 (72.41)	46 (55.42)	525 (44.57)	1,474 (54.59)
CRF01_AE	10 (32.26)	53 (21.46)	3 (10.00)	1 (16.67)	62 (21.23)	286 (22.02)	213 (36.54)	284 (27.84)	11 (12.64)	13 (15.66)	312 (26.49)	636 (23.56)
CRF08_BC	2 (6.45)	46 (18.62)	3 (10.00)	1 (16.67)	52 (17.81)	158 (12.16)	41 (7.03)	142 (13.92)	5 (5.75)	11 (13.25)	161 (13.67)	320 (11.85)
CRF55_01B	0 (0.00)	20 (8.10)	0 (0.00)	1 (16.67)	20 (6.85)	21 (1.62)	18 (3.09)	50 (4.90)	0 (0.00)	3 (3.61)	50 (4.24)	51 (1.89)
B	2 (6.45)	5 (2.02)	0 (0.00)	0 (0.00)	5 (1.71)	23 (1.77)	20 (3.43)	38 (3.73)	0 (0.00)	2 (2.41)	39 (3.31)	53 (1.96)
C	1 (3.23)	12 (4.86)	3 (10.00)	0 (0.00)	14 (4.79)	51 (3.93)	20 (3.43)	39 (3.82)	2 (2.30)	3 (3.61)	43 (3.65)	89 (3.30)
A	0 (0.00)	2 (0.81)	1 (3.33)	0 (0.00)	3 (1.03)	7 (0.54)	13 (2.23)	11 (1.08)	3 (3.45)	1 (1.20)	14 (1.19)	19 (0.70)
Others	1 (3.23)	6 (2.43)	0 (0.00)	0 (0.00)	6 (2.05)	17 (1.31)	20 (3.43)	32 (3.14)	3 (3.45)	4 (4.82)	34 (2.89)	58 (2.15)
Years												
2014–2015	0 (0.00)	6 (2.43)	0 (0.00)	0 (0.00)	6 (2.05)	74 (5.70)	79 (13.55)	90 (8.82)	1 (1.15)	0 (0.00)	90 (7.64)	97 (3.59)
2016–2017	1 (3.23)	6 (2.43)	0 (0.00)	0 (0.00)	6 (2.05)	29 (2.23)	96 (16.47)	105 (10.29)	2 (2.30)	0 (0.00)	106 (9.00)	123 (4.56)
2018–2020	14 (45.16)	134 (54.25)	18 (60.00)	0 (0.00)	157 (53.77)	683 (52.58)	143 (24.53)	195 (19.12)	11 (12.64)	1 (1.20)	208 (17.66)	370 (13.70)
2021–2022	7 (22.58)	61 (24.70)	8 (26.67)	0 (0.00)	70 (23.97)	290 (22.32)	170 (29.16)	381 (37.35)	45 (51.72)	41 (49.40)	462 (39.22)	1,223 (45.30)
2023–2024	9 (29.03)	40 (16.19)	4 (13.33)	6 (100.00)	53 (18.15)	223 (17.17)	95 (16.30)	249 (24.41)	28 (32.18)	41 (49.40)	312 (26.49)	887 (32.85)
ART regimen												
3TC+LPV+TDF	4 (12.90)	16 (6.48)	1 (3.33)	1 (16.67)	18 (6.16)	18 (1.39)	90 (15.44)	134 (13.14)	17 (19.54)	7 (8.43)	165 (14.01)	174 (6.44)
3TC+TDF+EFV	26 (83.87)	202 (81.78)	25 (83.33)	5 (83.33)	242 (82.88)	1,216 (93.61)	122 (20.93)	164 (16.08)	7 (8.05)	3 (3.61)	172 (14.60)	352 (13.04)
3TC+ZDV+EFV	1 (3.23)	13 (5.26)	3 (10.00)	0 (0.00)	15 (5.14)	15 (1.15)	46 (7.89)	50 (4.90)	1 (1.15)	0 (0.00)	51 (4.33)	59 (2.19)
3TC+ZDV+LPV	0 (0.00)	2 (0.81)	0 (0.00)	0 (0.00)	2 (0.68)	2 (0.15)	85 (14.58)	133 (13.04)	16 (18.39)	15 (18.07)	161 (13.67)	169 (6.26)
3TC+ZDV+NVP	0 (0.00)	14 (5.67)	1 (3.33)	0 (0.00)	15 (5.14)	48 (3.70)	66 (11.32)	90 (8.82)	4 (4.60)	2 (2.41)	100 (8.49)	142 (5.26)
3TC+DTG							32 (5.49)	69 (6.76)	7 (8.05)	9 (10.84)	83 (7.05)	221 (8.19)
3TC+DTG+LPV							0 (0.00)	7 (0.69)	0 (0.00)	1 (1.20)	8 (0.68)	8 (0.30)
TAF+FTC+BIC							65 (11.15)	172 (16.86)	14 (16.09)	17 (20.48)	194 (16.47)	843 (31.22)
3TC+TDF+DTG							43 (7.38)	127 (12.45)	13 (14.94)	23 (27.71)	161 (13.67)	481 (17.81)
TDF+3TC+ANV							34 (5.83)	74 (7.25)	8 (9.20)	6 (7.23)	83 (7.05)	251 (9.30)

### Prevalence of ART drug resistance

In this study, most NRTI-class drugs exhibited a high proportion of high-level resistance. Notably, PLWH with PDR and ADR exhibited the equivalent high-level resistance rate to lamivudine (3TC) and emtrictitabine (FTC), with respective value of 4.79% (X^2^ = 24.58, *P* < 0.001) and 25.04% (X^2^ = 433.94, *P* < 0.001). For NNRTIs, high proportions of potential low-level resistance were observed among individuals with PDR (*P* < 0.001). In contrast, the NNRTI-associated ADR was predominantly characterized by high-level resistance to nevirapine (NVP) and efavirenz (EFV), with corresponding rates of 35.23% (X^2^ = 319.13, *P* < 0.001) and 33.36% (X^2^ = 276.77, *P* < 0.001). PI-class and INSTI-class drugs mainly harbored potential low-level drug resistance in both PDR and ADR PLWH. For PIs, potential low-level drug resistance to nelfinavir (NFV) in PDR and ADR sequences were 8.90% (X^2^ = 18.24, *P* < 0.001) and 5.26% (X^2^ = 93.91, *P* < 0.001) respectively. Regarding to INSTI-related PDR and ADR, Elvitegravir (EVG) and raltegravir (RAL) exhibited the potential low-level resistance at frequencies of 1.03% and 3.65%, respectively (Figure 2). In terms of resistant drugs, the top three NNRTI drugs with PDR and ADR were NVP (76.03%; 82.09%), etravirine (ETR) (75.00%; 63.50%) and EFV (69.18%; 79.29%). For NRTIs, didanosine (ddI) exhibited the highest PDR rate (9.59%), whereas FTC had the highest ADR rate (46.86%). In PI drugs, NFV accounted for the highest proportion of PDR and ADR with 9.93% and 7.39%, respectively. The top three INSTIs with PDR and ADR were EVG (1.71%; 6.28%), RAL (1.71%; 6.28%) and cabotegravir (CAB) (1.03%; 2.89%) (Figure 3). In terms of HIV-1 subtypes with PDR, four NRTIs (3TC, FTC, ddI, ABC), five NNRTIs (DLV/DOR, EFV, NVP, ETR, RPV) and two PIs (NFV, TPV) showed significantly different prevalence (*P* < 0.05). The CRF07_BC subtype accounted for the highest proportion of NRTI and NNRTI-related PDR sequences, with the highest prevalence observed in 3TC (57.89%) (X^2^ = 20.21, *P* < 0.001), FTC (57.89%) (X^2^ = 20.21, *P* < 0.001), and EFV (48.51%) (X^2^ = 290.52, *P* < 0.001). In azidothymidine (AZT) drug with PDR, CRF01_AE percentage was highest at 42.86%. Furthermore, CRF01_AE was the predominant subtype for most PI-related PDR, while CRF07_BC dominated PDR resistance to NFV (68.97%) (X^2^ = 43.93, *P* < 0.001) and Tipranavir (TPV) (75.00%) (X^2^ = 45.58, *P* < 0.001). For INSTI-associated PDR, CRF07_BC subtype occupied the highest proportion in EVG (40.00%) and RAL (40.00%), while CRF01_AE and CRF08_BC showed the highest percentage in bictegravir (BIC) (50.00%) and dolutegravir (DTG) (50.00%). In NNRTI and NRTI drugs with ADR, significant differences in prevalence were observed among HIV-1 subtypes (*P* < 0.05). CRF07_BC was the predominant subtype, at 44.33% (EFV) (X^2^ = 1,281.88, *P* < 0.001), 43.33% (NVP) (X^2^ = 1,276.67, *P* < 0.001) and 40.51% (ETR) (X^2^ = 790.70, *P* < 0.001) for NNRTI; and 59.46% (EVG) (X^2^ = 156.92, *P* < 0.001), 59.46% (RAL) (X^2^ = 156.92, *P* < 0.001), and 28.00%(DTG) (X^2^ = 15.00, *P* = 0.037), for INSTI. Similarly, CRF07_BC dominated the subtype distribution for most NRTI drugs with ADR, while CRF01_AE was the dominant subtype for AZT resistance (35.90%) (X^2^ = 85.69, *P* < 0.001). In PI drugs with ADR, CRF07_BC percentage were highest at 76.12% (TPV) (X^2^ = 134.57, *P* < 0.001), follow by 72.22% (NFV) (X^2^ = 204.40, *P* < 0.001) (Figure 4).

**Figure 2:**
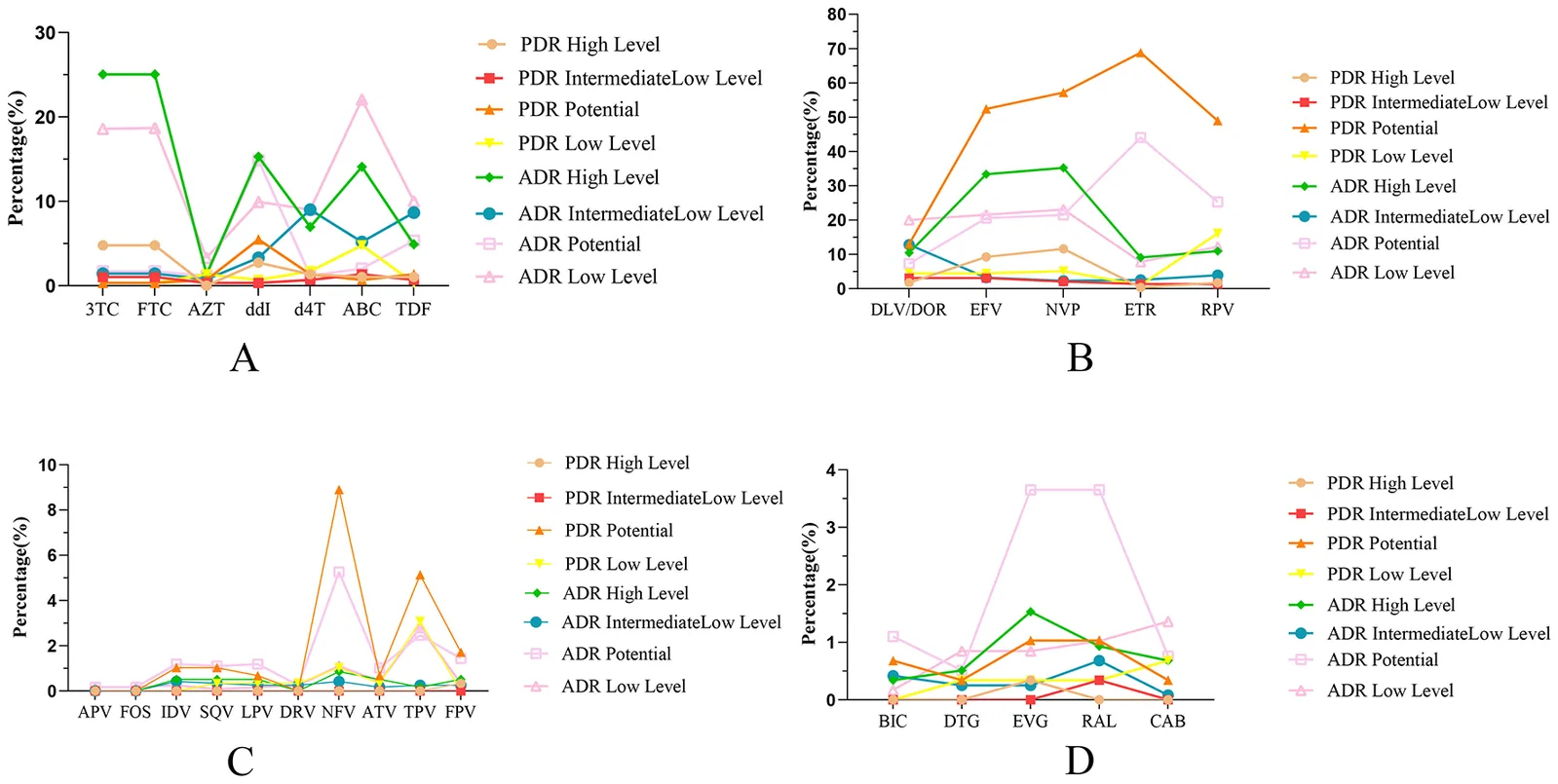
The drug resistance level. **(A)** NRTIs; **(B)** NNRTIs; **(C)** PIs; **(D)** INSTIs. 3TC, Lamivudine; FTC, Emtrictitabine; AZT, Azidothymidine; ddI, Didanosine; d4T, Stavudine; DLV/DOR, Delavirdine/Doravirine; EFV, Efavirenz; NVP, Nevirapine; ETR, Etravirine; RPV, Rilpivirine; APV, Amprenavir; FOS, Fosamprenavir; IDV, Indinavir; SQV, Saquinavir; LPV, lopinavir; DRV, Darunavir; NFV, Nelfinavir; ATV, Atazanavir; TPV, Tipranavir; FPV, Fosamprenavir; BIC, Bictegravir; DTG, Dolutegravir; EVG, Elvitegravir; RAL, Raltegravir; CAB, Cabotegravir.

**Figure 3:**
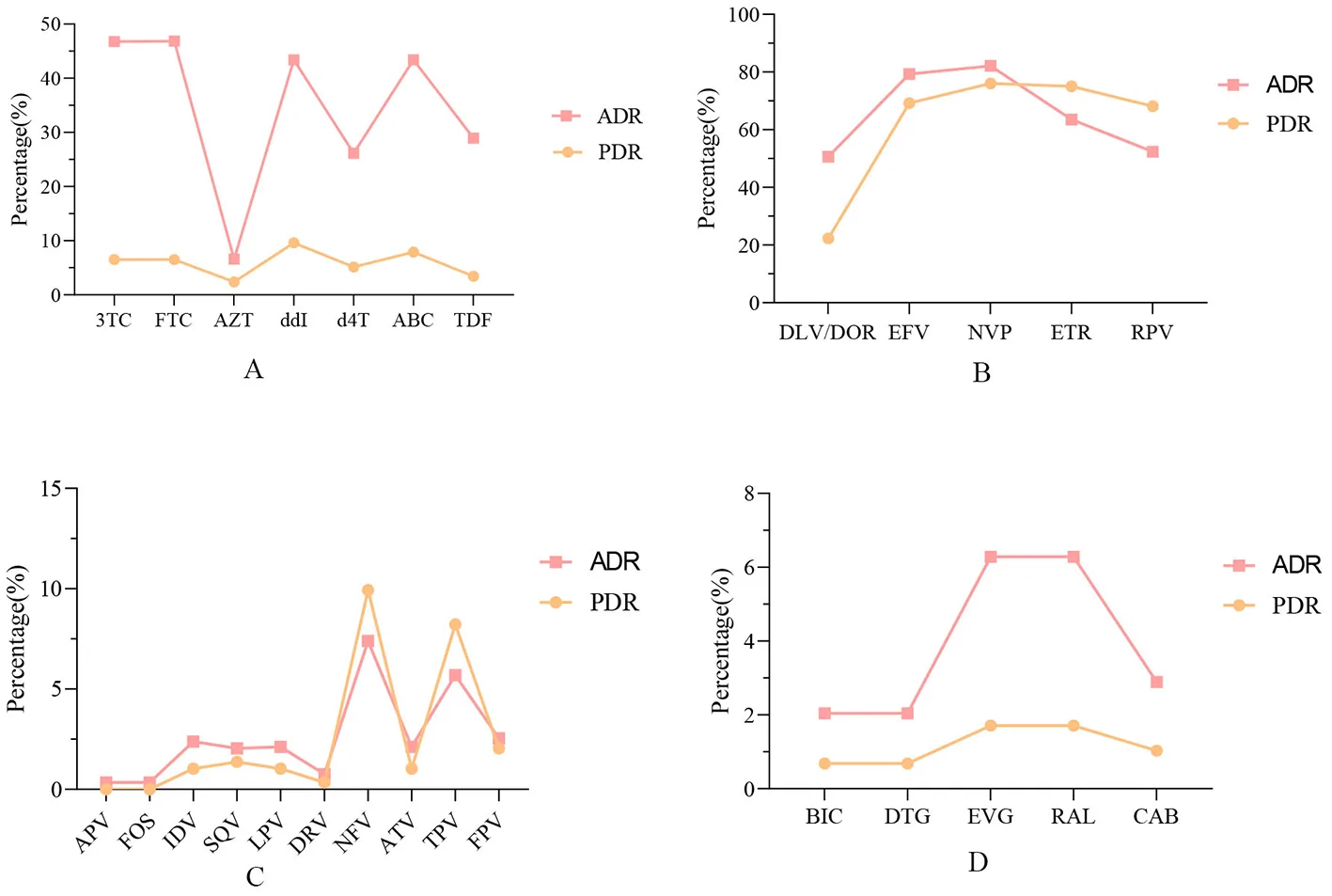
The proportion of PDR and ADR. **(A)** NRTIs; **(B)** NNRTIs; **(C)** PIs; **(D)** INSITs.

**Figure 4:**
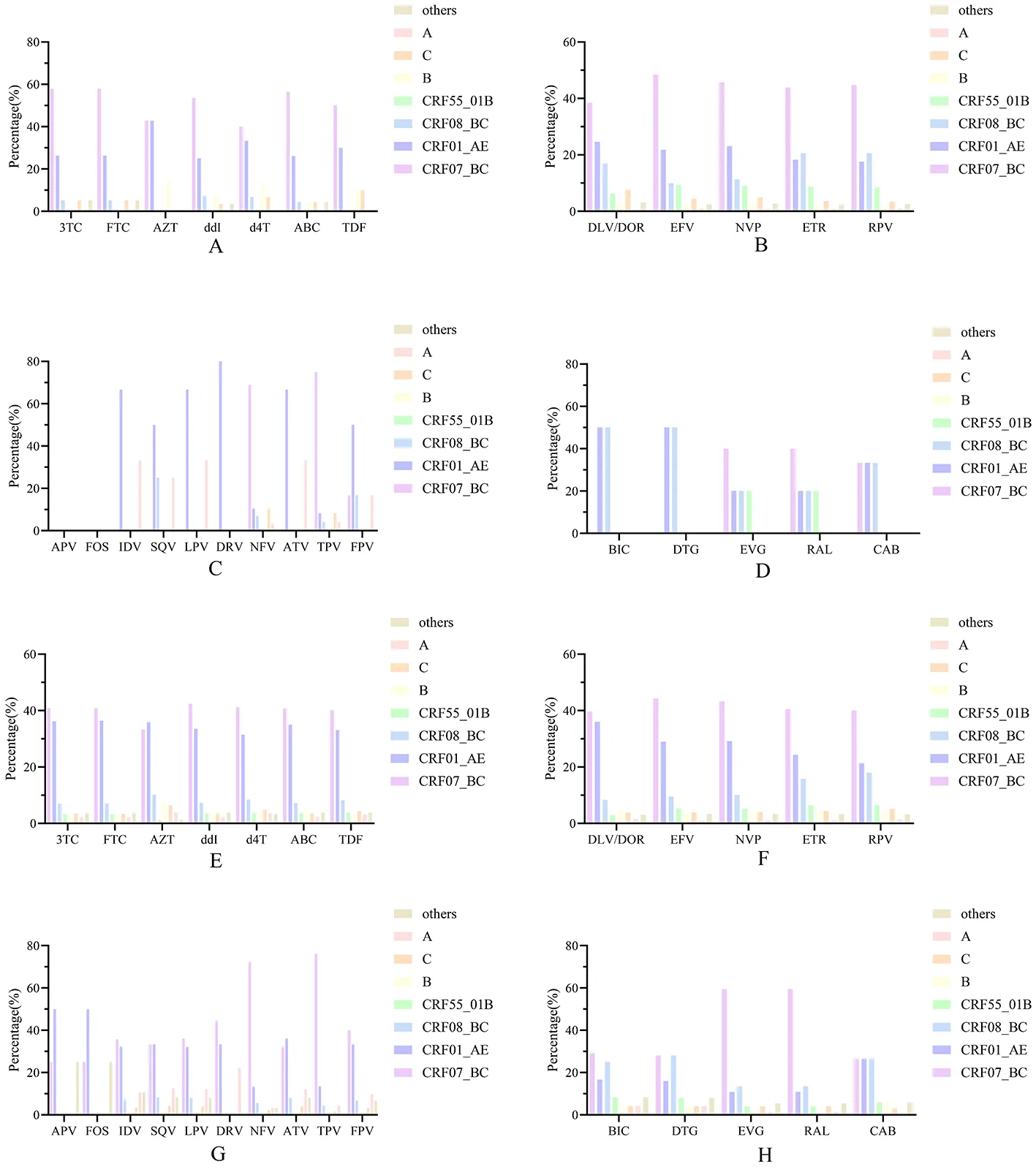
The proportion of PDR and ADR in different HIV-1 subtypes. **(A)** NRTIs with PDR; **(B)** NNRTIs with PDR; **(C)** PIs with PDR; **(D)** INSTIs with PDR; **(E)** NRTIs with ADR; **(F)** NNRTIs with ADR; **(G)** PIs with ADR; **(H)** INSTIs with ADR.

### Prevalence of drug resistance mutations

The proportions of the PDR and ADR mutations are shown in Figure 5. In NRTIs with PDR and ADR, the most common mutation was M184V at frequencies of 2.40% and 34.72%, respectively. In NNRTI with PDR, the highest mutation incidence of V179D was 32.53%, followed by V179E (14.73%) and K103E (8.56%). However, the most common NNRTI-associated ADR mutation was V179D (26.57%), followed by K103E (22.92%) and V106M (18.25%). The top PI-associated PDR mutations were K43T (4.11%) and Q58E (3.77%), whereas the most common ADR mutation was Q58E (3.06%). The highest prevalence of PI-associated PDR and ADR major mutation was M46I/L, at frequencies of 0.68% and 1.36%, respectively. All INSTI-associated PDR and ADR mutations identified in this study presented low prevalence. The most common PDR mutation was L74M (1.03%), followed by E157Q (0.68%). Regarding INSTI-associated ADR, E92Q (0.51%), Q148R (0.42%), and E138K (0.34%) were the top three major mutations, while the most prevalent minor mutations were E157Q (2.80%), A28T (1.19%), and L74M (1.02%).

**Figure 5:**
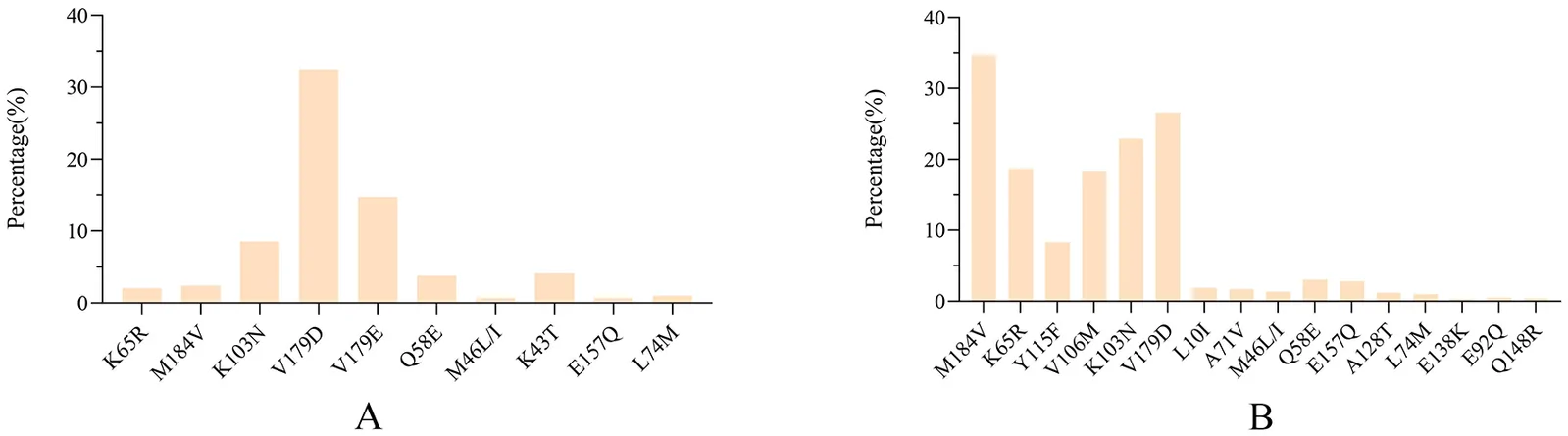
The proportions of the PDR and ADR mutations. **(A)** PDR; **(B)** ADR.

### Prevalence of drug resistance mutations in HIV-1 subtypes

Figure 6 illustrated the proportions of PDR and ADR mutations in different HIV-1 subtypes. For PDR sequences, four NNRTI-associated mutations (K103N, V179D, V179E, E138A) and one PI-associated mutation (M46L/I) exhibited significantly different prevalence among HIV-1 subtypes (*P* < 0.05). The predominated PDR mutations in the CRF_07BC subtype were K65R (3.08%) and M184V (3.08%) for NRTI; V179D (43.85%) (X^2^ = 23.61, *P* = 0.001) for NNRTI; K43T (9.23%) for PI; E157Q (1.54%) and L74M (1.54%) for INSTI. The top mutation in CRF01_AE subtype with PDR was T69N (6.54%) for NRTI; V179D (40.32%) for NNRTI and M46I/L (1.61%) for PI. In CRF08_BC subtype, the most common PDR mutation was E138A (X^2^ = 67.35, *P* < 0.001) at frequencies of 44.23%. Additionally, V179E (80.00%) (X^2^ = 62.20, *P* < 0.001) was identified as the predominant PDR mutation in the CRF55_01B subtype. Regarding ADR mutations, four NRTI-associated mutations (M184V, K65R, D67N, Y115F), five NNRTI-associated mutations (V106M, K103N, V179D, V179E, E138A), two PI-associated mutations (Q58E, M46I/L) and one INSTI-associated minor mutation (E157Q) showed significantly different prevalence among HIV-1 subtypes (*P* < 0.05). M184V was the most prevalent NRTI resistance mutation in all HIV-1 subtypes included in this study expect subtype A. For CRF01_AE, M184V exhibited the highest prevalence at 45.83% (X^2^ = 39.97, *P* < 0.001). In NNRTI-associated ADR mutations, the predominant mutations were V179D (32.57%) (X^2^ = 45.38, *P* < 0.001) in CRF07_BC, K103N (29.49%) (X^2^ = 21.20, 0 *P* < 0.001) in CRF01_AE, E138A (30.43%) (X^2^ = 250.00, *P* < 0.001) in CRF08_BC, and V179E (98.00%) (X^2^ = 422.35, *P* < 0.001) in CRF55_01BC, respectively. For PI-related ADR mutations, Q58E was the most common mutation in the CRF07_BC subtype, with a prevalence of 6.48% (X^2^ = 36.63, *P* < 0.001). M46I/L, as the major PI mutation, showed a relatively high prevalence in CRF01_AE (2.56%) (X^2^ = 19.85, *P* = 0.002). Consisted with the prevalence of the INSTI-associated PDR mutations, the ADR mutations also exhibited a low incidence. In CRF07_BC subtype, the most common INSTI-associated major mutation was E92Q (0.38%), followed by E138K (0.19%) and Q148R (0.19%); and the most common minor mutation was E157Q (5.14%), followed by A128T (2.48%) and L74M (0.38%). In CRF01_AE subtype, the two most prevalent major INSTI-associated ADR mutations were E92Q (0.64%) and Q148R (0.64%), while the top two minor mutations were L74M (1.60%) and E157Q (0.96%).

**Figure 6:**
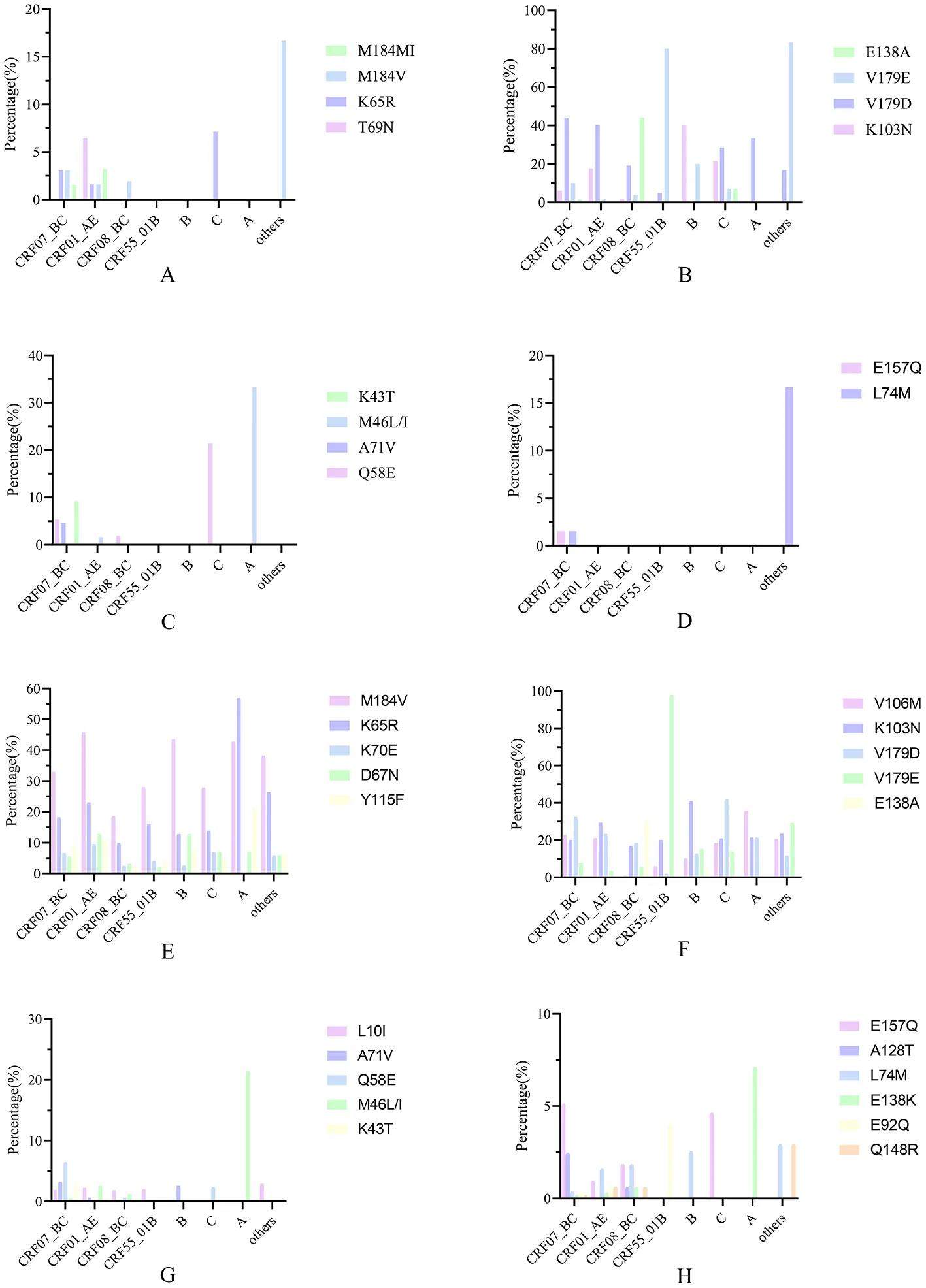
The PDR and ADR mutations proportions in different HIV-1 subtypes. **(A)** NRTIs with PDR; **(B)** NNRTIs with PDR; **(C)** PIs with PDR; **(D)** INSTIs with PDR; **(E)** NRTIs with ADR; **(F)** NNRTIs with ADR; **(G)** PIs with ADR; **(H)** INSTIs with ADR.

### Drug resistance analysis of ART regimen

Figure 7 showed the distribution of ART regimens in different study periods. For ART-naive PLWH, 3TC+TDF+EVF served as the primary initial ART regimen. Among ART-treated PLWH, 3TC+TDF+EVF remained the predominant ART regimen from 2014 to 2020; whereas TAF+FTC+BIC became the most common ART regimen during 2021 to 2024. All ART regimens with PDR exhibited the high proportion of NNRTI-class drug resistance. Specifically, in patients with PDR under 3TC+TDF+EFV regimen, the resistance frequencies to NNRTIs were 76.03% for NVP, 72.31% for ETR, 69.01% for EFV, and 64.05% for RPV. Correspondingly, all ART regimens with ADR showed a significantly higher proportion of NNRTI-class drug resistance (*P* < 0.001). For 3TC+TDF+EFV regimen with ADR, the resistance rates were 93.02% for NVP, 88.37% for EFV, and 59.30% for ETR. For TAF+FTC+BIC regimen with ADR, the prevalence of resistance to NVP, EFV, and ETR was 81.44%, 79.90%, and 71.65%, respectively (Figure 8). Figure 9 presented PDR and ADR mutations in different ART regimens. In PDR cases receiving 3TC+LPV+TDF and 3TC+TDF+EFV regimens, M184V was the most prevalent NRTI-associated mutation, at frequencies of 11.11% and 2.07% respectively. V179D mutation dominated NNRTI-related PDR in multiple traditional regimens, with high prevalence rates of 33.33% in 3TC+LPV+TDF regimen, 35.12% in 3TC+TDF+EFV regimen, and 33.33% in 3TC+ZDV+EFV regimen. In contrast, V179E was the predominant NNRTI-associated PDR mutation identified in patients under 3TC+ZDV+NVP regimen. In 3TC+TDF+EFV regime, the most common PI- and INSTI-associated PDR mutations were Q58E (4.13%) and L74M (1.24%) respectively. And in the major PI-associated mutation detected in this regimen, M46I/L (0.83%) had a relatively high prevalence. For the four NRTI-associated (M184V, K65R, K70E, D67N), two NNRTI-associated (V106M, V179D), two minor PI-associated (L10I, A71V), and two minor INSTI-associated ADR mutations, significant differences in prevalence were observed in ART regimens (*P* < 0.05). M184V was the dominant NRTI-associated mutation with prevalence rates of 48.26% in 3TC+TDF+EFV regimen and 25.26% in TAF+FTC+BIC regimen (X^2^ = 122.00, *P* < 0.001). V179D had a highest prevalence of NNRTI-associated mutations in 3TC+TDF+EFV (34.88%) and TAF+FTC+BIC (30.93%) (X^2^ = 48.21, *P* < 0.001). For PI-related ADR mutations in TAF+FTC+BIC regimen, K43T (3.61%) was the most common minor mutation, while M46I/L (1.03%) was the most common major mutation. In terms of INSTI-associated ADR mutations in TAF+FTC+BIC regimen, L74M, the minor mutation showed the highest prevalence, at frequency of 3.61% (X^2^ = 18.13, *P* = 0.004); and E92Q (1.03%) was the most common major mutation.

**Figure 7:**
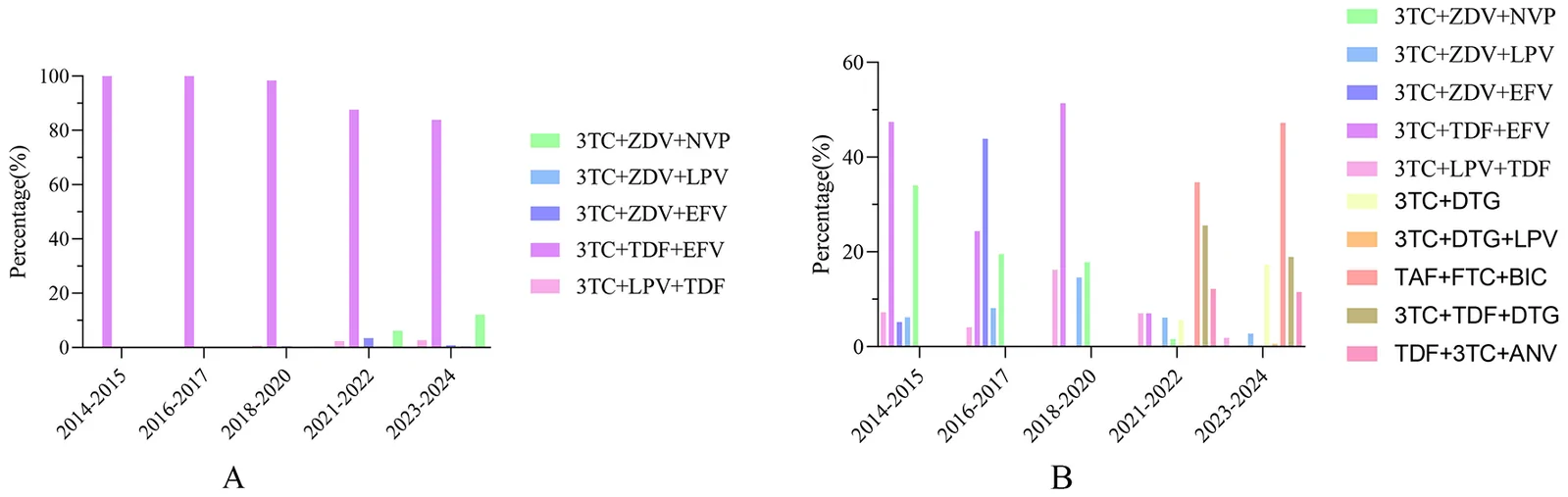
The proportion of the ART regimens in different periods. **(A)** In ART-naïve PLWH; **(B)** In ART-treated PLWH.

**Figure 8:**
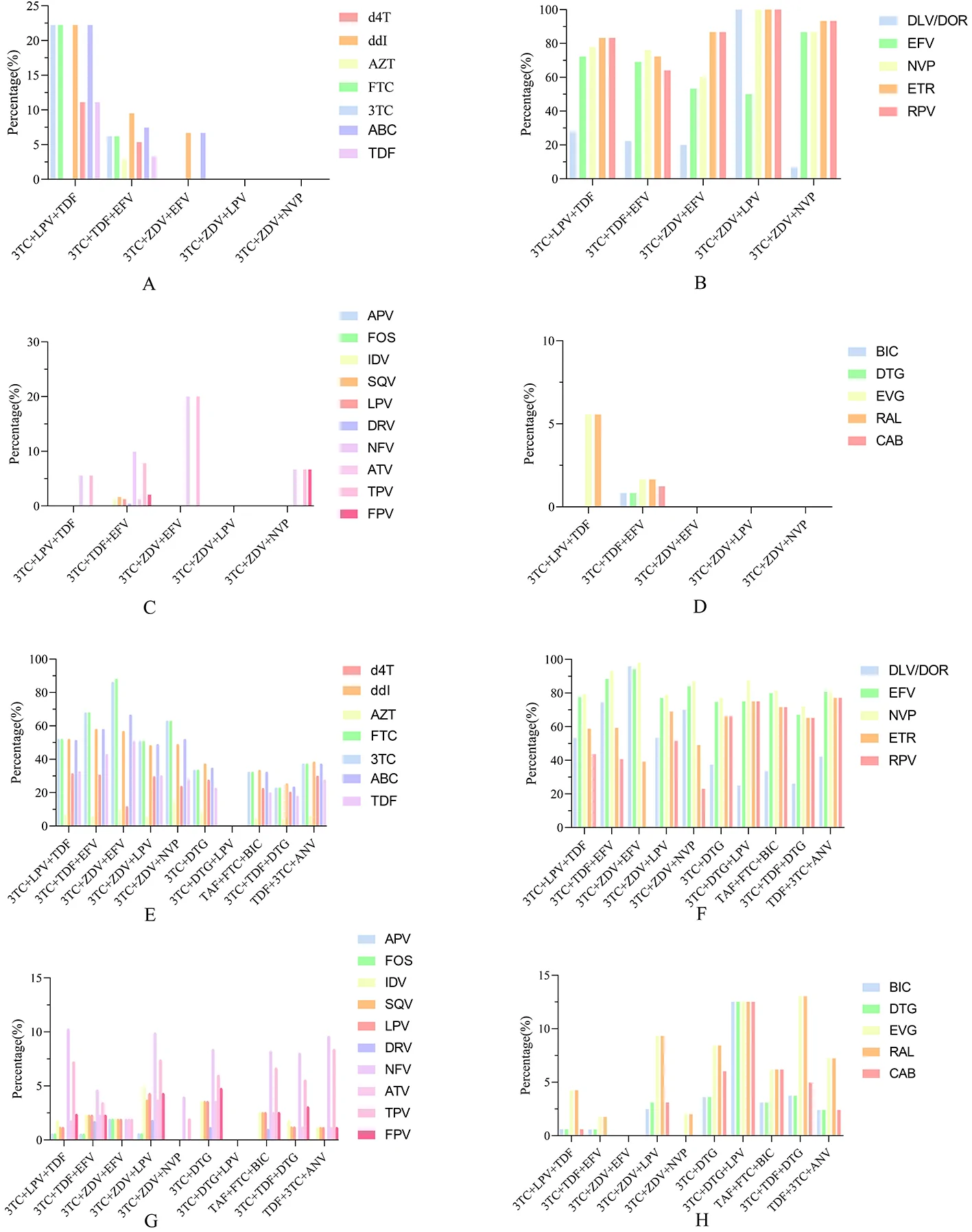
The frequencies of PDR and ADR in different ART regimens. **(A)** NRTIs with PDR; **(B)** NNRTIs with PDR; **(C)** PIs with PDR; **(D)** INSTIs with PDR; **(E)** NRTIs with ADR; **(F)** NNRTIs with ADR; **(G)** PIs with ADR; **(H)** INSTIs with ADR.

**Figure 9:**
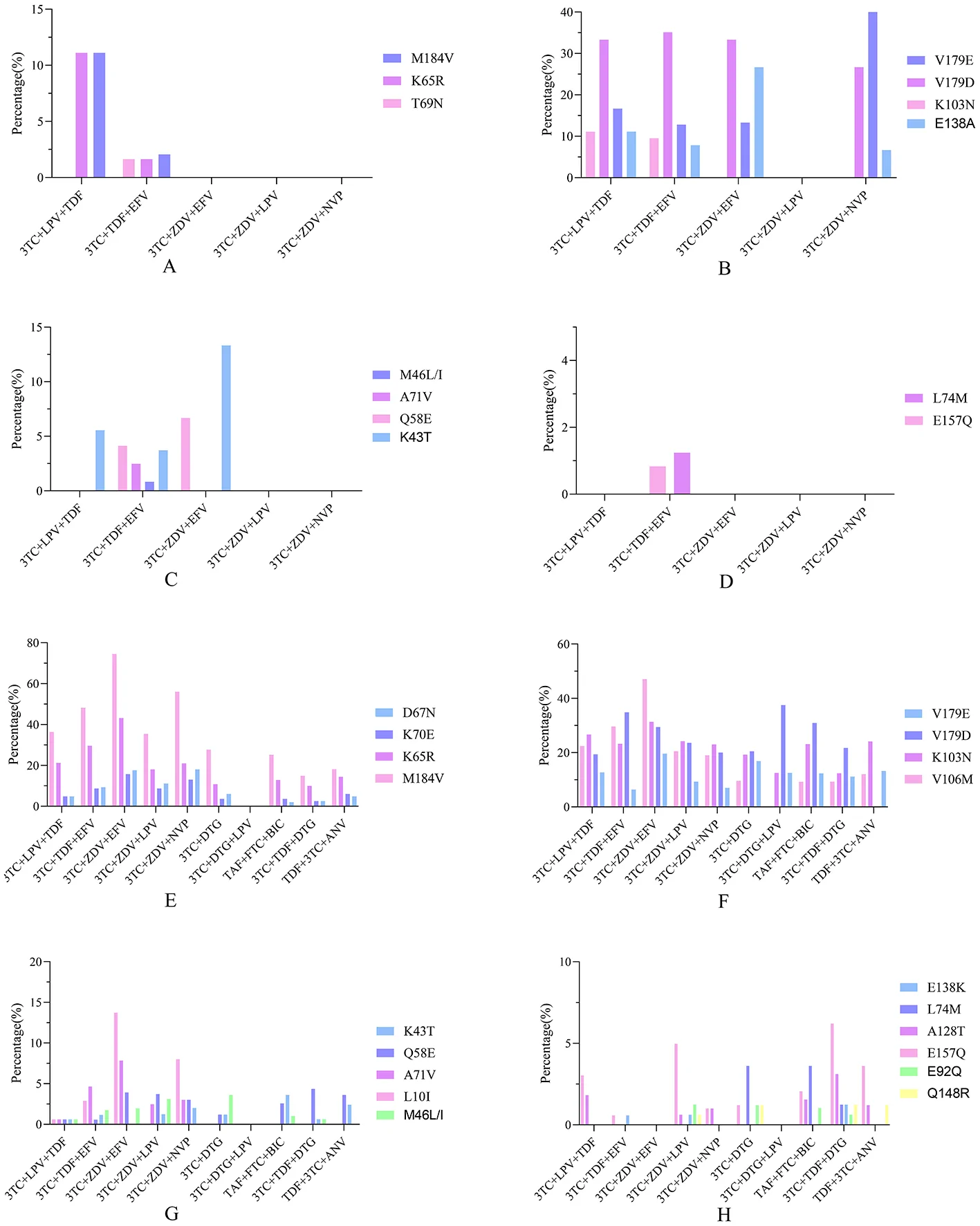
The PDR and ADR mutations proportions in different ART regimens. **(A)** NRTIs with PDR; **(B)** NNRTIs with PDR; **(C)** PIs with PDR; **(D)** INSTIs with PDR; **(E)** NRTIs with ADR; **(F)** NNRTIs with ADR; **(G)** PIs with ADR; **(H)** INSTIs with ADR.

### Risk factors for HIV drug resistance

To further explore the risk factors associated with HIVDR, univariable and multivariable logistic regression analyses were performed. As shown in Table 2, female (aOR = 1.50) and CRF08_BC subtype (aOR = 2.20) were the risk factors for HIVDR among ART-naive PLWH. For ART-treated PLWH, Compared with CRF01_AE (aOR = 1.40), CRF08_BC (aOR = 1.98), subtype B (aOR = 4.23), and subtype C (aOR = 1.71), CRF07_BC was associated with a significantly lower risk of HIVDR. Regarding to ART regimens, the dual NRTIs + NNRTI regimen conferred a higher risk of HIVDR than the NRTI + INSTI regimen (aOR = 0.38) and the dual NRTIs + INSTI regimen (aOR = 0.80), while demonstrating a lower risk than the dual NRTIs + PI regimen (aOR = 19.00).

**Table 2:** The risk factors of HIVDR.

	ART-naive PLWH					ART-treated PLWH				
Variable	HIVDR, *N*	Unadjusted OR	*P* value	Adjusted OR	*P* value	HIVDR, *N*	Unadjusted OR	*P* value	Adjusted OR	*P* value
Sex										
Male	219	Ref		Ref		893	Ref			
Female	73	1.44 (1.06, 1.96)	0.02	1.50 (1.01, 2.06)	0.01	285	1.08 (0.90, 1.29)	0.40		
Age (years)										
≤ 24	18	Ref				63	Ref		Ref	
25–49	143	1.04 (0.59, 1.84)	0.88			589	1.49 (1.06, 2.08)	0.02	1.50 (0.99, 2.26)	0.06
≥50	131	0.74 (0.42, 1.31)	0.30			525	1.03 (0.74, 1.44)	0.85	1.26 (0.83, 1.89)	0.28
CD4 + T cell count (cells/μL)										
<200	234	Ref				891	Ref		Ref	
200–500	52	0.99 (0.70, 1.39)	0.96			251	0.08 (0.67, 0.96)	0.02	1.00 (0.81, 1.23)	0.99
>500	5	0.82 (0.31, 2.19)	0.69			33	0.94 (0.59, 1.49)	0.80	1.03 (0.61, 1.73)	0.93
HIV subtype										
CRF07_BC	130	Ref		Ref		525	Ref		Ref	
CRF01_AE	62	1.29 (0.92, 1.81)	0.14	1.24 (0.89, 1.75)	0.21	312	1.74 (1.44, 2.10)	<0.001	1.40 (1.13, 1.74)	0.002
CRF08_BC	52	2.29 (1.56, 3.35)	<0.001	2.20 (1.50, 3.23)	<0.001	161	1.83 (1.44, 2.34)	<0.001	1.98 (1.51, 2.60)	<0.001
CRF55_01B	20					50				
B	5	1.30 (0.47, 3.55)	0.62	1.32 (0.48, 3.62)	0.60	39	5.04 (2.71, 9.36)	<0.001	4.23 (2.15, 8.22)	<0.001
C	14	1.76 (0.93, 3.36)	0.08	1.61 (0.84, 3.08)	0.15	43	1.69 (1.10, 2.60)	0.017	1.71 (1.06, 2.75)	0.05
A	3	3.50 (0.77, 15.80)	0.10	3.35 (0.74, 15.24)	0.12	14	5.06 (1.81, 14.13)	0.002	2.67 (0.86, 8.28)	0.09
Others	6	2.54 (0.92, 7.00)	0.07	2.67 (0.97, 7.37)	0.06	34	2.56 (1.50, 4.37)	0.001	2.24 (1.22, 4.12)	0.01
ART regimens										
Dual NRTIs +NNRI	272					406	Ref		Ref	
NRTI +INSTI						83	0.59 (0.43, 0.80)	0.001	0.62 (0.45, 0.85)	0.003
Dual NRTIs +INSTI						355	0.36 (0.30, 0.43)	<0.001	0.38 (0.31,0.46)	<0.001
Dual NRTIs +PI	20					326	18.80 (11.32, 31.21)	<0.001	19.00 (11.41, 31.65)	<0.001
NRTI+PI+INSTI						8				

## Discussion

In this study, the overall prevalence of PDR was 22.48%, with 2.39%, 19.01%, 2.31% and 0.46% exhibiting resistance to NRTIs, NNRTIs, PIs and INSTIs, respectively, which was higher than that reported in a previous report (15). However, the prevalence showed a fluctuating trend from 2014 to 2024. Although PDR prevalence showed only a slight decline from 2023 to 2024, the finding aligns with previous research indicating a rising trend in national PDR prevalence from 2013 to 2022 (9). Delayed HIV diagnosis, delayed ART regimen switching, insufficient supply and the high price of third-line ART drugs may contribute to an increasing temporal trend of PDR (4). These findings highlight the urgent need for intensified surveillance of drug resistance among newly diagnosed individuals. The total prevalence of ADR was 43.67%, which was slightly lower than that reported in previous studies (16). Additionally, the ADR prevalence to NRTIs, NNRTIs, PIs and INSTIs was 21.59%, 37.70%, 3.22% and 3.07%, respectively. However, the prevalence of NNRTI- and NRTI-associated ADR declined steadily from 2014 to 2024, in contrast to the rising trends observed for PI- and INSTI-related ADR. This discrepant temporal trend may be attributable to the widespread implementation of routine PDR testing and the growing clinical emphasis on standardized ART adherence management. Secondly, in Chongqing region, INSTI-class drug resistance testing has been gradually conducted since 2020, and was incorporated into routine clinical testing in 2022. These measures assist clinicians in selecting optimal ART regimens for PLWH and further underscore the necessity of continuous PDR and ADR monitoring. CRF07_BC, the most prevalent HIV-1 subtype in Chongqing, was first identified among injecting drug users in Yunnan province and has evolved into the dominant HIV variant among drug-using populations in Yunnan Province and Liangshan Prefecture over the past two decades (17). The widespread prevalence of CRF07_BC in Chongqing may be attributed to the geographical proximity to Yunnan Province and Liangshan Prefecture, as well as increased population mobility driven by rapid socioeconomic and transportation development. Similarly, the prevalence of CRF01_AE and CRF08_BC showed a temporal increasing trend. Currently, CRF01_AE has become the predominant variant circulating among men who have sex with men (MSM). And CRF08_BC was the predominant strain among heterosexuals and injecting drug users in Southern China, and heterosexual transmission has become the primary route of HIV infection nationwide (11, 18, 19). In recent years, heterosexual transmission has remained the dominant route of HIV infection in Chongqing, followed by MSM, which accounts for 19.5%. The MSM population has become one of the high-risk groups for HIV-1 infection and is characterized by high mobility, which partly accounts for the rising prevalence of CRF01_AE and CRF08_BC. (18).

In NRTI mutations associated with PDR and ADR, the most common mutation was M184V, which serves as a hallmark mutation conferring resistance to 3TC and FTC (20). The widespread use of 3TC and FTC within the first-line ART regimens also confirms the high-level resistance of 3TC and FTC in this study, consistent with the previous reports (13, 21, 22). Furthermore, different HIV-1 subtypes exhibit distinct patterns of drug resistance and mutations (23). For CRF07_BC, most NRTIs drugs had a high prevalence of PDR and ADR. K65R and M184V represented the most common PDR mutations in CRF07_BC, whereas T69N was the most prevalent mutation in CRF01_AE. K65R mutation confers intermiddle- or high-level resistance to TDF and ABC. The high frequency of K65R may be attributable to the dominant use of the first-line regimen 3TC+TDF+EFV in Chongqing (13, 14, 20). T69N mutation is frequently detected in patients exposed to thymidine analogs. Furthermore, thymidine analogs mutations (TAMs) are transmitted to HIV-uninfected persons (24). Accumulation of distinct TAM combinations confers varying degrees of resistance to AZT (25). The research in South Korea reported a progressive rise in T69N prevalence among patients receiving AZT for more than 48 months (26). When accumulated with other TAMs, this would lead to enhanced resistance to AZT, TDF, d4T, ABC and ddI (24, 27). Previous studies have indicated that in CRF01_AE, thymidine analogs drugs preferentially selected TAM-2 pathway over the TAM-1pathway. Nevertheless, the selected pathway was reversed among individuals receiving AZT-based treatment regimens (28). This phenomenon partially accounts for the substantial AZT resistance burden observed in CRF01_AE strains with PDR and ADR. In NRTI-associated ADR mutations, M184V was the predominant mutation in all HIV-1 subtypes except subtype A. In addition, for CRF07_BC and CRF01_AE with ADR, the incidence of D67N and K70R mutations was second to that of M184V and K65R mutations. TAMs were frequently observed in patients who have failed first-line ART regimen containing TDF. This correlation is supported by evidence showing that the accumulation of three or more TAMs diminishes viral susceptibility to TDF and drives the emergence of broad-spectrum HIV-1 multi-drug resistance (29). However, a study demonstrated that TAF was effective at suppressing viruses in patients harboring TAMs, and was further effective up to six TAMs combined with M184V mutation (25). This therapeutic advantage explains why the TAF+FTC+BIC regimen has become the dominant ART regimen for ART-treated patients in Chongqing.

In this study, the prevalence of PDR and ADR was primarily driven by NNRTI-class drug. Consistent with the previous study, patients with ADR displayed high-level resistance to NVP and EFV, whereas only low-level resistance was detected among individuals with PDR (15, 16). NNRTIs possess a relatively low genetic barrier to resistance, as a single mutation is sufficient to confer drug resistance (20). In addition, NNRTI-based first-line ART regimens were widely used in China, which may promote the emergence and persistence of drug-resistance mutations and allow these variants to persist stably in the viral population (11). Accordingly, these drug-resistant mutations are easily detected. Our study found that the most common mutation with PDR was V179D, followed by V179E and K103E. V179D, K103E and V106M were the most frequently observed in ADR sequences, all of which confer drug resistance to NVP and EFV (13, 30). Notably, E138A was the dominant mutation in the CRF08_BC subtype, which is consistent with findings from a previous study conducted in Nanning (11). E138A is predominantly clustered in Yunnan and exhibits the high rate of resistance to RPV, which may result in virological failure in patients receiving RPV-based regimens and induce cross-resistance to NVP, EFV and ETR (20, 31). Additionally, six polymorphic mutations (S16C, K102Q, T200A, V179E, I202V, T200M) were co-variated with E138A. Collectively, these finding suggest that E138A is the signature mutation of CRF08_BC. Given the expanding scale of heterosexual transmission, the prevalence of the CRF08_BC subtype has been continuously increasing. Therefore, we should pay more attention to E138A mutation in CRF08_BC subtype.

In this study, the most common PI-associated major mutation in PDR and ADR sequences was M46I/L, while the most common minor mutation was Q58E, followed by K43T. Overall, PI-class drugs exhibited a low prevalence of drug resistance, which may be attributed to the high resistance barrier, delayed clinical introduction in Southwest China and the primary clinical application as the second-line regimen (13, 32). M46I/L confers PI resistance individually or in combination with other mutations, and is associated with the susceptibility reduction to ATV, FPV, IDV, LPV and NFV (5, 18). In this study, M46I/L demonstrated the highest prevalence among patients receiving 3TC+ZDV+LPV regimen. This finding may be attributable to the HIV treatment guidelines that recommended LPV as a preferred ART drug switch following NNRTI -based treatment failure in second-line regimens. Previous study found that M46I/L mutation is predominantly observed in CRF01_AE-infected MSM population (33), which is consistent with our observation of a high prevalence of M46I/L in the CRF01_AE subtype. Although PI-class drugs are predominantly employed in the second-line regimens, their widespread use may lead a rising prevalence of PI-associated drug resistance in the future. In addition, with the continuous growth of MSM population in Chongqing, sustained surveillance of M46I/L transmission within this high-risk group is required.

In 2018, China proposed INSTI-based regimens as the recommended first-line ART treatment. Furthermore, the Chinese Guidelines for the Diagnosis and Treatment of HIV/ADIS (2024) recommended two NRTIs (TDF/ABC+3TC/FTC; TAF+FTC) combined a third-class drug as the standardized first-line ART regimens. Specifically, TAF+FTC+BIC is identified as the preferred first-line regimen (34). TAF+FTC+BIC regimen has been recognized globally and domestically as one of the first-line treatments characterized by high efficacy, low toxicity, and a high barrier to drug resistance. Accordingly, it has emerged as the dominant therapeutic option for re-treatment patients in the Chongqing. In this study, the most common INSTI mutation in PDR sequences was L74M, followed by E157Q. The predominant major mutation in ADR sequences was E92Q, followed by Q148R and E138K, while the most common minor mutation with ADR were E157Q, A128T and L74M. Only one patient with PDR harboring both V179D and L74M mutations exhibited potential low-level resistance to CAB, whereas other patients carrying isolated L74M mutation showed no INSTI resistance. This may be because the presence of NNRTIs- and/or NRTIs-class PDR may increase the risk of developing resistance to INSTIs (35). Previous research has demonstrated that minor mutation can indirectly facilitate the emergence of INSTI resistance by amplifying the resistance effect of major mutations or enhancing viral fitness (36). Specifically, E157Q may promote drug resistance by altering the structural stability of the integrase or affecting its DNA-binding capacity (37). Our study further verified that E157Q mutation conferred low-level potential resistance to both EVG and RAL and was the most prevalent INSTI mutation among PLWH with CRF07_BC, which was consistent with previous studies (38, 39). E92Q is a drug resistance-associated polymorphic INSTI mutation that modulates HIV-1 enzyme activity and replicative capacity, while enhancing the fitness and virulence of HIV-1 strains against INSTIs (38). In our study, E92Q mutation conferred intermediate to high-level resistance to EVG and RAL, as well as potential low-level to low-level against second-generation INSTIs (BIC, DTG and CAB). E138K is a non-polymorphic mutation that frequently co-occurred with Q148R mutation. The co-occurrence induces high-level resistance to RAL and EVG, while intermediate reductions in susceptibility to DTG and BIC (35). Our study further confirmed this conclusion that patients carrying both E138K and Q148R mutations develop high-level resistance to INSTI drugs. Although INSTI drugs are not yet currently added to the national free antiretroviral medication catalog, they have been covered by the national health insurance, enabling the majority of patients to afford them without financial burden. The delayed introduction of INSTIs and the short duration of clinical application in Chongqing contributed to the low prevalence of INSTI resistance. Nevertheless, with the widespread use of INSTIs, efforts should be directed toward strengthening INSTI resistance monitoring and optimizing INSTI-based regimens.

We further explored the risk factors associated with ADR and PDR. Our results revealed that among ART-naive PLWH, female and CRF08_BC subtype were independent risk factors for drug resistance, which is consistent with previous studies (15). Although CRF55_01B was excluded from the risk factor analysis due to its extremely sparse distribution (21 samples, 20 of which were HIVDR-positive), the high HIVDR prevalence among this population should not be overlooked. CRF55_01B subtype, a recombinant subtype formed by the recombination of CRF01_AE and B, originated in MSM group (40), while CRF08_BC subtype predominates among heterosexual individuals and injecting drug users. With the continuous expansion of heterosexual transmission and the increase of MSM group, PLWH with CRF55_01B and CRF08_BC would have a higher risk on drug resistance. Therefore, among newly diagnosed PLWH, we should pay more attention on drug resistance for females, CRF55_01B and CRF08_BC populations, and routine resistance screening should be strengthened in these high-risk populations. However, among ART-treated PLWH, CRF01_AE, CRF08_BC, subtype B, subtype C and treatment with the dual NRTIs+PI regimen were identified as independent risk factors of HIVDR. Ongoing improvements in regional transportation, socioeconomic development and the growth of labor-importing population may further drive an increasing trend in HIVDR prevalence in Chongqing. Additionally, PI-based regimens are commonly recommended as second-line therapy for patients who experience virological failure on first-line NNRTI-based regimens. Consequently, patients receiving the 2NRTIs + PI regimen are likely to have harbored pre-existing NRTI- and NNRTI-related resistance mutations. Although these pre-existing mutations are not reversed after transition to a PI-based regimen, ongoing viral replication under suboptimal adherence or insufficient regimen efficacy may facilitate the emergence of additional PI-associated resistance mutations. This cumulative process may contribute to the substantially increased risk of HIV drug resistance observed in patients receiving the 2NRTIs + PI regimen. Collectively, these findings highlight the critical need for resistance genotyping prior to therapy switch.

Our study still has some limitations. First of all, samples were recruited from only one hospital that was the designated hospital in Chongqing responsible for admitting and treating PLWH and many cases with incomplete clinical information were excluded. The single-center sampling strategy may limit the generalizability of our findings and fail to fully reflect the overall prevalence of ADR and PDR. Secondly, potential selection bias may have resulted in an overestimated PDR prevalence compared with previously reported. Thirdly, our study did not include the transmission routes and ART medication adherence, which may have overlooked the potential influence of these factors on drug resistance.

In conclusion, the study is the first to analysis the comprehensively prevalence of ART drugs (NRTI, NNRTI, PI and INSTI) with PDR and ADR in Chongqing. The epidemic character of PDR and ADR varied significantly depending on the distinct HIV-1 subtypes and ART regimens. With the growth of population mobility and the widespread use of INSTI-based regimens, the epidemic characteristic of PDR and ADR are expected to become more diverse in the future. Therefore, regular monitoring of drug resistance and the timely assessment and adjustment of ART regimens are crucial for the prevention and control of HIVDR.

## Data Availability

The datasets generated for this study are not publicly available due to privacy-ethical restrictions. Requests to access the datasets should be directed to the corresponding author.
