# Supplementary material for: Prevalence and transmission of pretreatment drug resistance and acquired drug resistance among people living with HIV-1 in Chongqing, China, 2014–2024
Source: Front Public Health. 2026 Sep 16;14:1923017. doi: 10.3389/fpubh.2026.1923017 (PMC13623912; doi:10.3389/fpubh.2026.1923017)
Supplement: Supplementary file 1 [file Table_1.docx]

Table S1 The prevalence of PDR and ADR between2014 and 2024.

|  | ART-naive PLWH | | | | | ART-treated PLWH | | | | | |
| --- | --- | --- | --- | --- | --- | --- | --- | --- | --- | --- | --- |
|  | 2014-2015  % | 2016-2017  % | 2018-2020  % | 2021-2022  % | 2023-2024  % | 2014-2015  % | 2016-2017  % | 2018-2020  % | 2021-2022  % | 2023-2024  % |  |
| NRTIs | 0.00 | 3.45 | 2.05 | 2.41 | 4.04 | 81.44 | 78.05 | 38.65 | 13.90 | 10.71 |  |
| NNRTIs | 8.11 | 20.69 | 19.62 | 21.03 | 17.94 | 92.78 | 85.37 | 52.70 | 31.15 | 28.07 |  |
| PIs | 0.00 | 0.00 | 2.64 | 2.76 | 1.79 | 1.03 | 1.63 | 2.97 | 3.68 | 3.16 |  |
| INSTIs | 0.00 | 0.00 | 0.00 | 0.00 | 2.69 | 0.00 | 0.00 | 0.27 | 3.35 | 4.62 |  |
| HIVDR | 8.11 | 20.69 | 22.99 | 24.14 | 23.77 | 92.78 | 86.18 | 56.22 | 37.78 | 35.17 |  |
